# Differentiation and Propagation Potential of *Arnica montana* L. Achenes as a Consequence of the Morphological Diversity of Flowers and the Position of Flower Heads on the Plant

**DOI:** 10.3390/plants11243424

**Published:** 2022-12-08

**Authors:** Piotr Sugier, Anna Rysiak, Danuta Sugier, Krystyna Winiarczyk, Dan Wołkowycki, Aleksander Kołos

**Affiliations:** 1 Department of Botany, Mycology and Ecology, Institute of Biological Sciences, Maria Curie-Skłodowska University, 19 Akademicka Street, 20-033 Lublin, Poland; 2 Department of Industrial and Medicinal Plants, University of Life Sciences in Lublin, 15 Akademicka Street, 20-950 Lublin, Poland; 3 Department of Cell Biology, Institute of Biological Sciences, Maria Curie-Skłodowska University, 19 Akademicka Street, 20-033 Lublin, Poland; 4 Department of Forest Environment, Institute of Forest Sciences, Bialystok University of Technology, 45E Wiejska Street, 15-351 Białystok, Poland

**Keywords:** *Arnica montana*, achene, glandular trichomes, morphological traits, reproductive characteristics

## Abstract

*Arnica montana* L. is a very important medicinal plant and simultaneously a European endemic endangered plant species. The morphological features and details of seed development and achene variability are poorly recognized. The aim of this study was to determine the impact of the achene position in the infructescence and the location of the inflorescence on the plant on the (i) morphological characteristics and germination ability of achenes, and (ii) recruitment of seedlings and their biometric features. Infructescences containing fully ripe achenes were randomly collected from *A. montana* individuals for the measurements and the germination experiment. Scanning electron microscopy, fluorescence microscopy, and light microscopy were used for characterization of flowers and achenes. The morphological traits of achenes and reproductive characteristics of *A. montana* were determined by the position of the achenes in the infructescence and the location of the inflorescence on the plant. The surface of arnica achenes is equipped with non-glandular and glandular trichomes, which is very rarely presented in species of the family Asteraceae. It is possible that the fluid-containing glandular trichomes are a source of essential oils. The peripherally located achenes were longer, thinner, and lighter. They were characterized by lower embryo weight, lower embryo/achene weight ratio, and lower germination capacity in comparison to the centrally located ones. The results presented in this article fill the gap in the knowledge of the morphology of achenes and the biology of the species, and provide information that can help in breeding programs, active protection, and field cultivation.

## 1. Introduction

The reproductive traits of vascular plants decline with time, and architectural constraints or resource limitation is responsible for the seasonal decreases in flower size, inflorescence size, and seed weight. Seeds or fruits that develop from early-pollinated flowers have access to a larger portion of the resource pool and are bigger. In turn, diaspores produced later are necessarily smaller due to the depleted resources [1]. Within an inflorescence, the available resources for the flowers and fruits seem to be unevenly distributed and vary according to their position in the inflorescence/infructescence [2,3]. The reproductive traits and fertility rates usually decrease from proximal flower positions to distal positions within the inflorescence [2,3,4,5]. This positional pattern of flower and fruit traits is characteristic e.g., for the capitulum inflorescence in the Asteraceae family [3]. Outer fruits within capitula are usually larger than inner fruits [6,7,8,9,10]. This positional variation is explained by competition between flowers for resources [2,3,4]. In the present paper, we intend to investigate whether *Arnica montana* (Asteraceae) exhibits such a variation in achene traits.

The formation of a capitulum inflorescence with two different types of florets is an interesting issue in floral biology and evolution. A frequent arrangement of the Asteraceae capitulum consists of peripherally located ray florets, which are highly specialized in pollinator attraction, and disc florets which serve the reproductive function [11,12]. Peripheral florets can be strongly zygomorphic (usually female), whereas disc florets are mainly actinomorphic (usually bisexual), which has important consequences for the evolution of reproductive biology [13,14,15,16,17,18]. The variation in the florets and, subsequently, in the fruit size and shape may also have profound ecological implications. Plants which exhibit a broad range of fruit sizes would be dispersed by a greater diversity of fruit consumers than plants which produce a narrow variety of fruit sizes [19]. Moreover, the achene weight and the achene and pappus shape play an important role in spatial diaspore dispersion, which is a requirement for establishment of new populations [20].

The mountain arnica (*Arnica montana* L.) is an endangered medicinal plant species. It is endemic for Europe; in natural habitats, it grows in heathlands and pine forests [21,22,23,24]. The organs of this species are characterized by the presence of valuable secondary metabolites, including sesquiterpene lactones, flavonoids, terpenoids, phenolic acids, and essential oils [25,26,27,28,29,30]. Flower heads, leaves, rhizomes, and roots are a rich source of raw material characterized by antibacterial, antifungal, antiseptic, anti-inflammatory, antioxidant, and antitumor properties [28,29,31,32,33,34,35,36,37]. Finally, *A. montana* achenes are a rich source of interesting molecules characterized by antioxidant and anticancer activity [29,38].

The possibilities for cultivation and reintroduction, and thus conservation, of arnica depend on the quality of achenes, which is measured by their germination capacity and vitality [28,29,30,39,40,41,42,43,44,45]. It has been analyzed in many field and greenhouse experiments and in the natural habitat using propagules from both cultivation and natural habitats [39,40,46,47]. Some limited aspects of the *A. montana* achene morphology were described by Aiello et al. [39] as an element of the use of wild populations in field cultivation. Achene traits and relationships between achene quality and dispersal distance, and the flying capability of plumed achenes in a wind-tunnel experiment were presented by Strykstra [20]. However, despite the importance of *A. montana* as a medicinal crop and a simultaneously protected species, the morphological characteristics and fundamental features of its reproductive biology, including details of seed development and achene variability, are poorly recognized. The results presented in this article fill the gap in the knowledge of the morphology and biology of the species and provide information that can help in breeding programs, active protection, and field cultivation. Therefore, the aim of this study was to determine the impact of the achene position in the infructescence and the location of the inflorescence on the plant on the (i) morphological characteristics and germination ability of achenes, and (ii) recruitment of seedlings and their biometric features.

## 2. Results

### 2.1. Morphological Observations of A. montana Achenes

**Figure 1:**
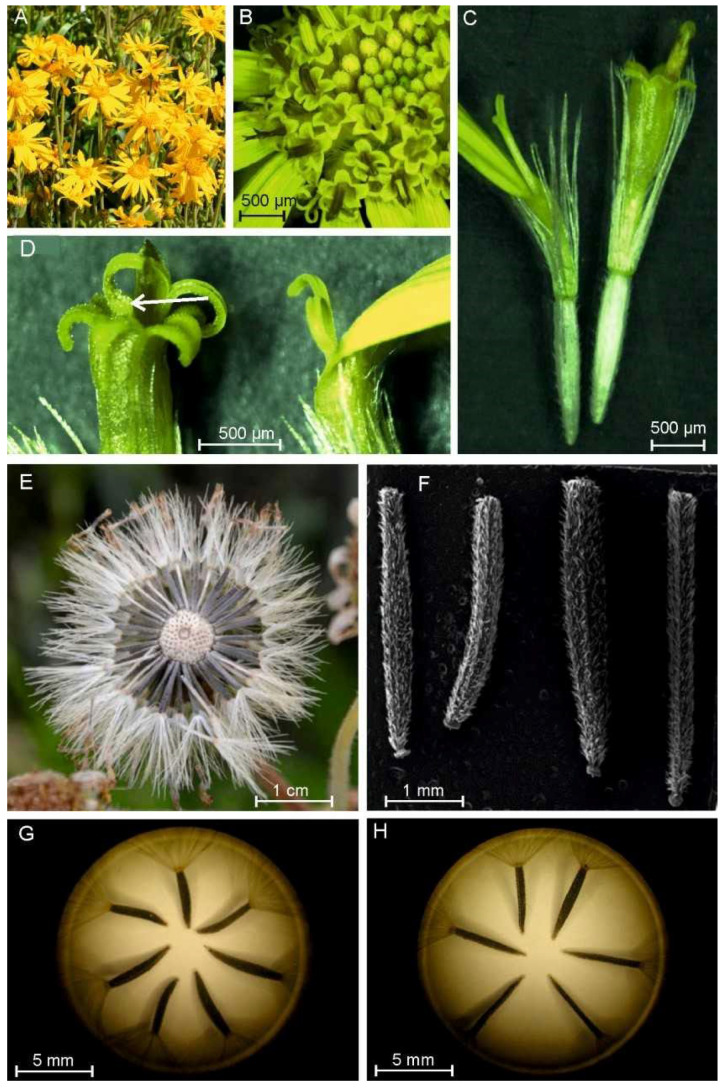
Specimens of *Arnica montana* L. in the full flowering phase (**A**). *A. montana* inflorescence at the anthesis stage; centripetal direction of opening of individual flowers in the inflorescence (**B**). Morphological diversity of flowers in the inflorescence: left—ligulate ray floret; right—tubular disc floret (**C**). Top part of the pistil in the two types of flowers with visible pollen grains (see arrow) deposited on the stigma surface (**D**). Achenes in infructescence—dead seeds are white, while viable seeds are dark grey (**E**). Morphological diversity of achenes: achenes obtained from ray ((**F**)—left site, (**G**)) and disc florets ((**F**)—right site, (**H**)).

The arnica flowers formed a single head inflorescence with peripherally located yellow female ray florets serving as an attractant. The central part of the head receptacle was occupied by tightly packed hermaphrodite disc florets. The disc florets had stamens located in the central part, which were fused with anther heads and formed a tube around the style. There were a greater number of disc florets in the head. The ray florets exhibited zygomorphic symmetry, while the disc florets were actinomorphic. The anthesis stage lasted approximately 7 days and was characterized by centripetal opening of individual florets (Figure 1A–D).

The morphological studies of achenes were conducted using 25 randomly selected mature infructescences. The achenes in the infructescence differed, i.e., the white ones were sterile non-viable seeds (Figure 1E), whereas the dark grey seeds were normally developed. The achenes produced by the arnica disc florets and ray florets differed in their shape and size (Figure 1F). The seeds in the ray florets were shorter and had shorter pappus elements (Figure 1G,H).

**Figure 2:**
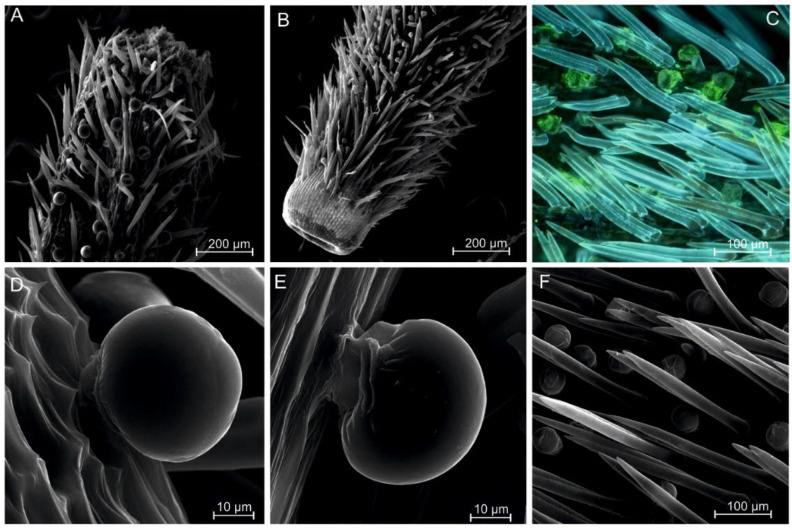
Micromorphology of the surface of arnica achenes from two zones: distal (**A**) and proximal (**B**). Two types of trichomes on the pericarp surface observed in FM. Lignin shows blue autofluorescence in two-celled, sharp-edged non-glandular trichomes, while glandular trichomes emit green fluorescence indicating the presence of cellulose (**C**). Two morphological and functional types of trichomes covering the pericarp: sharp-edged, elongated non-glandular trichomes and spherical glandular trichomes (**F**). Two-celled glandular trichomes composed of a stalk attached to the pericarp wall and a large spherical apical cell (**D**,**E**).

The arnica pericarp was covered with numerous morphologically diverse trichomes (Figure 2). Two types of trichomes: non-glandular and glandular were observed on the entire surface of the pericarp greater number of these structures were located in the distal (Figure 2A) and proximal (Figure 2B) parts of the pericarp. The non-glandular trichomes were composed of two cells and were sharply pointed towards the flight apparatus. The glandular trichomes were morphologically diverse: they were composed of a stalk attached to the pericarp wall and a large spherical apical head cell (Figure 2D,E). They were embedded deeper and adhered closely to the undulating surface of the pericarp. It was found that many of the spherical apical cells of these trichomes in mature achenes collapsed or had ruptured walls, indicating the release of their contents.

The FM observations conducted with the use of appropriate lignin-detecting filters revealed intense autofluorescence of the trichome walls. Blue autofluorescence was shown in the two-celled pointed non-glandular trichomes, indicating the presence of lignin in the cell wall of these structures. In contrast, the glandular trichomes emitted green fluorescence, indicating the presence of cellulose (Figure 2C). All trichomes were produced by a single-layer epidermis. The glandular trichomes were morphologically diverse; they were composed of an apical bulbous or spherical head and stalk cells (Figure 2D,E). They consisted of viable cells with intensely stained cytoplasm and a nucleus. The trapezoidal cells of the stalk base were covered by double or single layers of small cells. The upper part of the achene was equipped with modified pappus. The flight apparatus consisted of many massive elements. A single element of the flight apparatus was composed of several dozen strongly elongated, pointed, and tightly adherent cells.

### 2.2. Infructescence on the Main Stem vs. Infructescence on the Branch Stem

**Figure 3:**
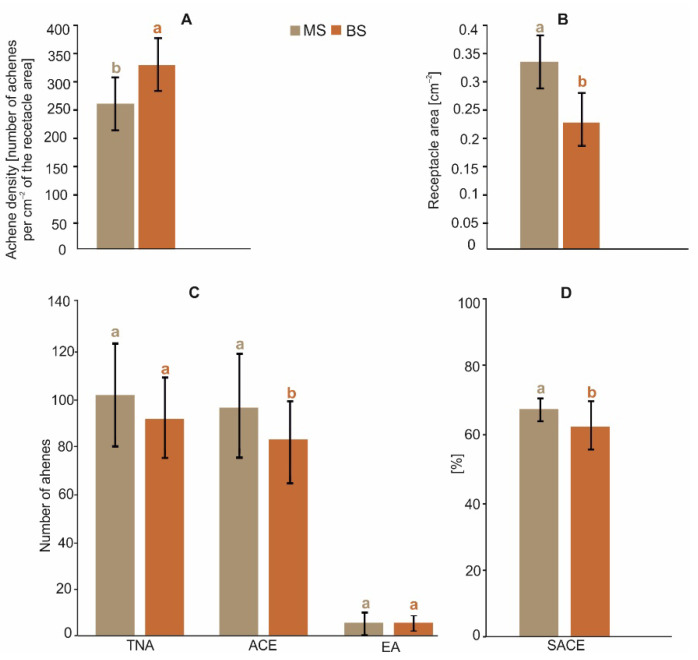
Comparison of achene density (**A**), receptacle area (**B**), number of achenes (**C**), and percent share of achenes (**D**) in two positions of *A. montana* flower heads on the plant (mean ± SD). MS—main stem; BS—branch stem; TNA—total number of achenes; ACE—number of achenes containing a developed embryo; EA—number of empty achenes; SACE—percent share of achenes containing a developed embryo. Different lowercase letters indicate significant differences between the means of two groups according to the *t*-test (*p* < 0.05).

The results of the statistical analyses showed a significant impact of the position of the infructescence on the plant on the number of achenes containing a developed embryo (t = 2.61, *p* < 0.05) (ACE), the percent share of achenes containing a developed embryo (t = 2.41, *p* < 0.05) (SACE), the receptacle area (t = 5.03, *p* < 0.001) (RA), and the achene density (t = −5.01, *p* < 0.001) (AD), but not on the total number of achenes (t = 1.84, *p* = 0.072) (TNA), and the number of empty achenes (EA) (t = −1.93, *p* = 0.059). The later developing inflorescence/infructescence on the branch stem was smaller and had a lower number of ACE, SACE, RA, and AD (Figure 3). The higher AD value in the infructescence on the branch stem indicates greater competition for resources initially between flowers and, later, between fruits. In consequence, we observed a lower SACE value (90.4%) in relation to infructescences located on the main stem (94.9%) in the absence of differences between these two analyzed groups in the case of TNA.

**Figure 4:**
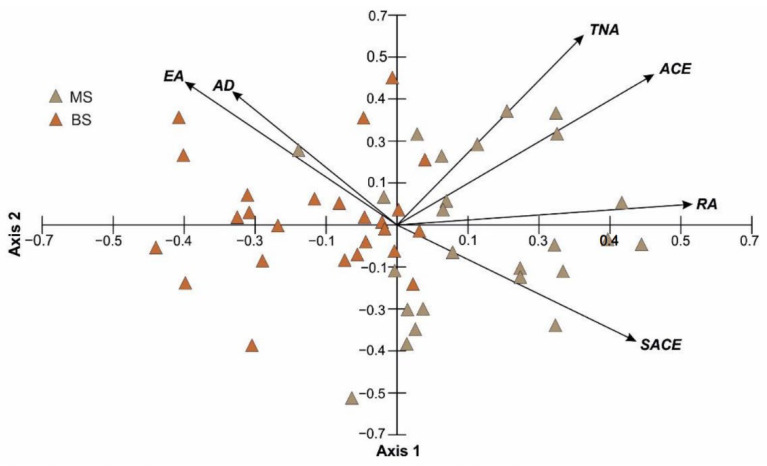
Results of PCA based on the characteristics of infructescences from the main stem and infructescences from the branch stem. MS—main stem; BS—branch stem; TNA—total number of achenes; ACE—number of achenes containing a developed embryo; EA—number of empty achenes; SACE—percent share of achenes containing a developed embryo; RA—receptacle area; AD—achene density.

Figure 4 shows the results of the PCA ordination on the basis of six characteristics of 25 infructescences located on the main stems, and 25 infructescences located on the branch stems. The two PCA axes explained 97.79% of the total variance, and they are sufficient to describe the analyzed samples (Table 1). The eigenvalues of the first (2.35), second (2.26), and third axis (1.25) indicate the presence of three gradients, within which the samples are differentiated in terms of the TNA, ACE, AD, SACE, RA, and AD values. The TNA, AC, SACE, and RA parameters are clearly positively correlated with the first axis, whereas EA and AD are negatively correlated. The TNA, ACE, EA, and RA traits are positively correlated with the second axis, whereas SACE is negatively correlated. The variation between the group of infructescences located on the main stems and the group of infructescences located on the branch stems is evident as well. Two groups can be distinguished in the ordination space of PCA. The infructescences located on the main stems are characterized by the highest values such parameters as TNA, AC, SACE, and RA, and the lowest values of EA and AD in relation to infructescences located on the branch stems (Figure 4, Table 1).

### 2.3. Morphometric Characteristics of Achenes

The results of the statistical analyses showed a significant impact of the achene position within the infructescence and the position of the infructescence on the plant on the majority of achene traits, such as the length of the achene with pappus (LAP), length of the achene without pappus (LA), pappus length (PL), width of the achene (WA), thickness of the achene (TA), and the achene weight (AW) (Table 2). The effect of the interaction of the achene position within the infructescence and the position of the infructescence on the plant was not confirmed statistically, only in the case of PL. In turn, in the case of the shape index (I), there was only an impact of the achene position within the infructescence.

**Figure 5:**
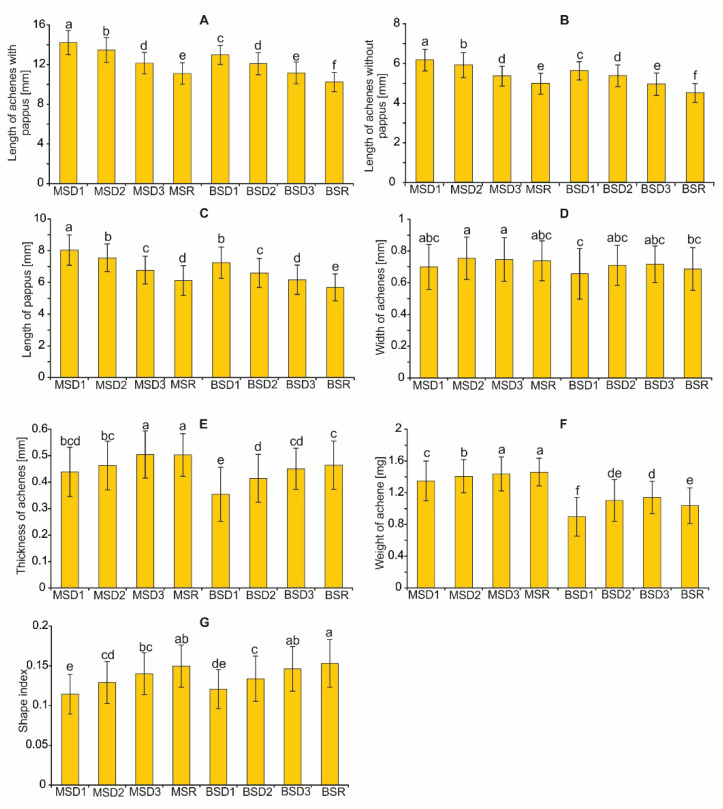
Morphological characteristics of *A. montana* achenes (**A**–**G**) from the four positions within the infructescence, and the two positions of the infructescence on the plant. MS—main stem; BS—branch stem; D1–D3—achenes produced by disc florets; R—achenes produced by ray florets. Different lowercase letters indicate significant differences according to the Tukey test (*p* < 0.05).

The values of such characteristics as LAP, LA, and PL increased from the proximal to distal area of the infructescence in the case of infructescences taken from both the main stem and the branch stem (Figure 5). The range of the LAP was from 11.10 mm to 14.21 mm, and from 10.25 mm to 12.98 mm for the main stem and the branch stem, respectively. The variations in LA ranged from 4.98 mm to 6.17 mm, and from 4.51 mm to 5.63 mm for the main stem and the branch stem, respectively. The PL values for achenes taken from the main stems and the branch stems ranged from 6.12 mm to 8.04 mm, and from 5.69 mm to 7.25 mm, respectively. The WA parameter in the compared groups was similar; nevertheless, the lowest value of this feature was determined for achenes from the upper part of the infructescence located on the branch stem (0.65–0.75 mm). In turn, the highest TA value was found for achenes produced by the ray florets (0.50 mm) and those produced by the disc florets (0.35 mm) situated peripherally on the receptacle. The AW value of all achene categories distinguished on the main stem ranged from 1.35 mm to 1.46 mm and was higher in relation to the achene groups distinguished in the infructescences taken from the branch stems. Among the analyzed factors, only the achene position within the infructescence showed a significant impact on the shape index (Table 2). The values of this parameter decreased from the outer to inner infructescence part.

The two-way ANOVA results showed a statistically significant impact of the achene position of the infructescence on the plant on the pericarp weight (PW) (Table 3) and on the embryo weight/achene weight (EW/AW) ratio. In turn, the achene position within the infructescence determined the embryo weight (EW), germination capacity (G), and EW/AW. The interaction of the analyzed factors was confirmed in the case of PW and G.

### 2.4. Germination, Survival and Seedling Characteristic

**Figure 6:**
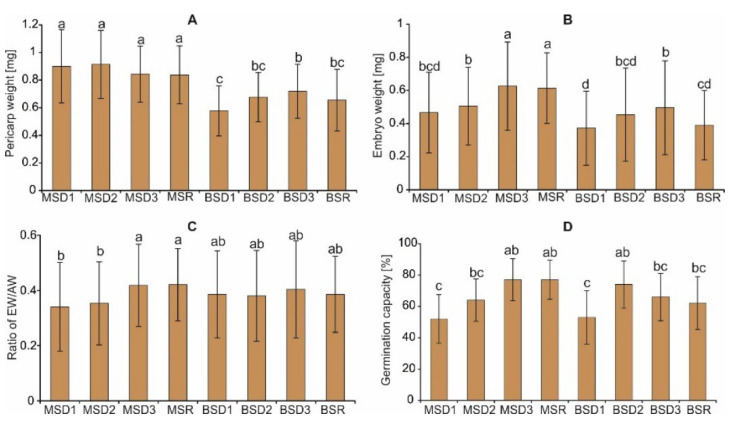
Comparison of *A. montana* seed characteristics (**A**–**C**) and germination capacity (**D**) of achenes from the four positions within the infructescence and the two positions of the infructescence on the plant. MS—main stem; BS—branch stem; D1–D3—achenes produced by disc florets; R—achenes produced by ray florets. Different lowercase letters indicate significant differences according to the Tukey test (*p* < 0.05).

The highest EW, EW/AW, and G values, i.e., 0.62 mg and 0.42, and 77%, respectively, were obtained in the case of achenes produced by the disc florets from the periphery of the receptacle (D3) and achenes produced by the ray florets (R) from infructescence on the main stem (Figure 6B–D). Achenes from the upper part of the receptacle produced by the disc florets (D1) of the branch stem were characterized by the lowest PW (0.58 mg) and EW (0.37 mg) values (Figure 6A,B). The lowest germination capacity was noted in the case of achenes from the upper part of the infructescence, as in the case of the main stem and the branch stem (Figure 6D). However, it should be emphasized that the achenes produced by the disc florets from the periphery of the receptacle (D3) and achenes produced by the ray florets (R) did not differ in the features discussed.

**Figure 7:**
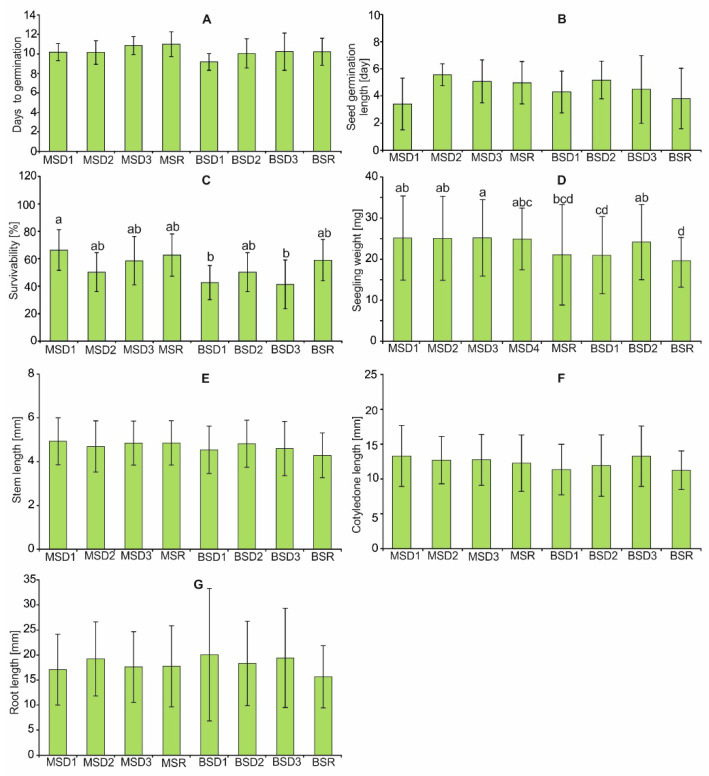
Days to germination, length of germination, and survival (**A**–**C**) and seedling measurements (**D**–**G**) of the four positions of achenes within the infructescence and the two positions of the infructescence on the plant. MS—main stem; BS—branch stem; D1–D3—achenes produced by disc florets; R—achenes produced by ray florets. Different lowercase letters indicate significant differences according to the U Mann–Whitney test (*p* < 0.05).

Statistically significant differences between the compared groups in the case of seedling weight (H = 20.83, *p* < 0.01) (Figure 7D) and survivability (H = 20.28, *p* < 0.01) (Figure 7C) were confirmed. The mean weight of seedlings developed from achenes taken from the infructescence of the main stem was similar among the distinguished categories and ranged from 24.90 mg to 25.14 mg. The seedling weight was significantly lower in the case of seedlings developed from achenes produced by the ray florets located on the capitula of the branch stem (19.62 mg). Such parameters as days to germination (H = 13.94, *p* = 0.052), (Figure 7A), seed germination length (H = 9.90, *p* = 0.194), (Figure 7B), stem length (H = 12.19, *p* = 0.094), (Figure 7E), cotyledon length (H = 13.46, *p* = 0.053), (Figure 7F), and root length (H = 5.41, *p* = 0.610) did not differ between the groups (Figure 7G).

## 3. Discussion

The observations of the arnica achenes showed that the pericarp cover was uneven, notched, and had numerous depressions. On this surface, morphologically diverse trichomes were present. A very large group was composed of secretory trichomes. The glandular trichomes were more varied, but all had a fluid-filled head at the apex. In angiosperms, trichomes may occur on leaves, petals, stems, petioles, peduncles, and seed coats, depending on the species. Trichomes are outgrowths of protodermal origin that can exist in all morphological parts of a plant [48,49]. Such morphological and mechanical features of trichomes as the density, size, shape, and surface texture, and their orientation may affect many aspects of plant physiology and ecology [48]. Trichomes can form at various stages of organ development. Some trichomes fall off the plant while others may remain until the plant senesces. Dead trichomes may still be involved in water absorption or seed dispersal [50]. The role of glandular trichomes is to produce a secretion that forms a continuous layer on the surface of the plant. They play an important role in plant defense against herbivores [51]. The active substances contained in these structures exhibit biological activities similar to those of insecticides and herbicides [52,53]. Previous studies have shown that arnica achenes are a source of an essential oil with anticancer activity [29]. It is possible that the fluid-containing trichomes are the source of the essential oil. However, confirmation of this thesis requires separate research.

The observation of the arnica achenes showed the presence of another type of these structures, i.e., mechanical/non-secretory trichomes, which were pointed and directed towards the distal part. Non-secretory trichomes serve different functions: they act as a mechanical barrier against insects [54,55], reflect light ([56,57], reduce transpiration [58,59], constitute hydrophobic layers for water droplets [56], and help plants survive. It should be emphasized that trichomes present on organs related to generative reproduction (flowers, fruits, and seeds) are more diverse and play a different role than trichomes located on the vegetative plant parts [60]. It has been reported that the hairs present on the petals of *Gossypium hirsutum* L. are necessary to maintain the correct shape of flower buds. Thanks to the mechanical entanglement of hairs from adjacent corolla petals, the proper architecture of the cotton flower is preserved. The silencing of the master regulator of petal trichome development caused flower buds to have abnormal shapes, exposing the developing anthers and pistils to desiccation damage [61]. In this context, the presence of so many different trichomes on the surface of arnica achenes and their role in the propagation cycle of this species are intriguing. In many angiosperms, the fruit is the unit of dispersion/germination rather than the seed. Therefore, the pericarp and the structures on its surface are extremely important in the spread and anchoring of diaspores and in seed germination. Ripe arnica achenes are dispersed by wind and gravity, and trichomes together with pappus significantly increase the dispersal distance, adhesion to soil particles, and the mass of water taken up and humidity. On the other hand, the presence of such a large number of trichomes in arnica achenes significantly increases the contact of their surface with the soil.

In the present study, the value of TNA per infructescence was 102 on the main stem and 92 on the branch stem, respectively. Maurice et al. [22] reported TNA values of 41.7 and 16.0 per infructescence for natural colline populations and montane populations, respectively, in Western Europe. This very large difference is related to the existence of very favorable habitat conditions during cultivation and the lack of competition from other plants, compared to natural populations. The mean achene weight presented in our study was in the range from 0.8 to 1.4 mg. These data are similar to the values of 1.4 mg and 1.2 mg reported for the colline and montane populations in Western Europe [22].

In Europe, some studies investigated the possibility of cultivation of *A. montana* L. In research on a natural population originating from the Carpathians, Ukraine, Balabanova et al. [62] showed germination capacity ranging from 75% to 79%. A field trial conducted by Aiello et al. [63] compared two wild accessions of mountain arnica collected in the province of Trento, Italy, and showed mean germination capacity in the range from 79.3% to 93.0%. In turn, Ukrainian and German arnica populations [64] exhibited a share of viable embryos of 68.97% and 63.46%, respectively. This indicates that similar germination capacity to our results. In the present study, the values of this parameter were clearly similar in the case of the peripheral achenes of the infructescence on the main stem. However, the germination capacity of the centrally located achenes within the infructescence on the main stem and the branch stem was lower.

In the case of natural arnica populations, the germination capacity, seed weight, and percentage of seeds containing a developed embryo are positively correlated with the population size depending on the environmental condition [21]. It is not excluded that the presented differences in the case of TNA per infructescence, achene weight, and germination are a result of the impact of many factors, e.g., different habitat conditions, species interrelations, and lack of competition in the field conditions. In the case of the field cultivation, the habitat conditions are optimal; therefore, in addition to generative propagation, more intensive vegetative propagation is observed [41,42], which is significantly limited in natural conditions [65,66]. Thus, in cultivation conditions, there is a division of resources between generative and vegetative propagation. It is not excluded that the genetic factor may also play a role.

In the case of some Asteraceae plant species, achene morphology affects the time of seedling emergence [67]. This dependence was not confirmed in the case of *A. montana*. Variations in morphological and functional characteristics of flowers grouped in inflorescences are commonly observed [68,69]. The resource competition hypothesis postulates that the variations in morphological and functional characteristics of flowers are attributable to competition among the ovaries of an inflorescence for a limited amount of resources [70,71,72,73,74,75]. In consequence, fruits initiated early and located peripherally and close to the source of nutrients and have the ability to assimilate more resources than fruits located more distally and initiated later [73].

Some studies have investigated relationships between the reproductive success and the within-inflorescence spatial position and time of flower opening (early or late). Plant species with acropetal or basipetal inflorescence development have higher fruit and seed set in early-opening flowers than in late-opening flowers [76,77,78,79,80]. In the case of the mountain arnica, the dynamics of flowering can also be observed in the development of the main and branch stems, the production of inflorescences on the branch stems, and the flowering inside the capitulum; hence, the lower radicle area, the higher number of empty achenes, and the higher percent share of embryo-containing achenes in the infructescence of the branch stem in relation to the main stem.

According to the non-uniform pollination hypothesis, the relatively low fruit or seed sets of certain flowers on the inflorescence may be attributable to variations in pollen receipt over the inflorescence flowering period (insufficient quantity or quality of pollen) [81,82,83,84]. It should be emphasized that the activity of pollinators is determined by the weather, i.e., its course, or, rather, the number of sunny days and their occurrence during the arnica flowering period, which lasts about 1 month [85]. They determine the reproductive success of flowers on inflorescences of both the main stem and branch stems. We observed the dynamics of flowering within arnica capitula during the study in the last years. The anthesis stage lasted approximately 7 days, and the direction of development of individual flowers was centripetal. In addition, inflorescences on branch stems are formed much later than inflorescences on the main stems. It is therefore likely that the flowers of the lateral capitula may be pollinated to a lesser extent. The species is highly self-incompatible [65], and is visited by many different insect groups, but predominantly by hoverflies and various bees [66].

The present results show that there is a significant intra-infructescence variation in the traits of *A. montana* achenes. The patterns of the variation in most variables show a decline from the lateral to central position. The decline was observed in the infructescence of the main stem and the branch stem. Such an intra-inflorescence variation is a common pattern in plants [5,68,70,76,77,82,86,87,88,89]. Although most studies have been focused on raceme-type inflorescences, similar patterns have been described in species with dichasium-type inflorescences as well [78,89]. Intra-inflorescence variation was also detected in components of reproductive success (e.g., fruit maturation and seed set); for example, in *Lavandula stoechas*, early-opening flowers were characterized by higher fruit set than later opening flowers [87].

Seed mass is regarded as an important aspect of the reproductive strategy [89]. In the literature, there are examples of plant species where the seed mass has an effect on germination and survival of seedlings and seedling performance [90,91,92,93]. In natural plant populations, large seed mass confers an advantage, principally in conditions where resources are scarce [90,94,95]. In the present study, the experimental conditions for germination were optimal and the resources were not limited. Therefore, no significant correlations were found between achene weight and values of the other seedling parameters.

In the present study, the mean embryo weight/achene weight ratio ranged from 0.33 to 0.41, whereas Strykstra et al. [20] reported a value of 0.6 in a natural mountain arnica population. Such a wide difference may reflect different habitat conditions. In the case of field conditions, no competition for resources and higher investment of arnica individuals in vegetative propagation were observed [42].

In the present study, the values of such characteristics as the achene weight, embryo weight, pericarp weight, and pappus length are similar to data presented from a natural population [20]. *A. montana* achenes are adapted to wind dispersal, thus the achene shape and mass and the pappus size play a key role in spatial dispersion. Strykstra et al. [20] conducted an experiment on the flying capability of plumed achenes in a wind-tunnel and showed a correlation between the dispersal distance and the achene quality in this species. Additionally, the germination capacity and seedling quality declined considerably with the increasing flying capability of the achenes. The author showed that only low-quality achenes reached larger distances. In the present study, the morphological traits of the achenes were shown to be dependent on the location within the infructescence and the position of the infructescence on the plant. In the infructescence, the achene quality was observed to change in the centripetal direction. The achenes in the infructescence center were characterized by low quality, i.e., the lowest achene mass, achene thickness, embryo weight, and achene germination capacity, which made the achene more able to fly.

## 4. Materials and Methods

### 4.1. Research Material

Fifty infructescences located on the main stems and fifty infructescences located on the branch stems containing fully ripe achenes intended for the research were randomly collected from fifty 3-year-old *A. montana* fruiting individuals growing on experimental plots (collection of the Department of Industrial and Medicinal Plants, University of Life Sciences in Lublin). The seed material was divided into two equal parts.

### 4.2. Scanning Electron Microscopy (SEM)

Samples for SEM observations were prepared according to the method described by Talbot and White [94]. Fresh material was fixed by immersion in methanol for 10 min and rinsed in ethanol. The material prepared in this way was dried in a CO_2_ atmosphere, sprayed with gold, and viewed in an LEO1430VP electron microscope with a 15k acceleration potential. Documentation was made using INCA-Mapping software (Billerica, MA, USA). The samples were analyzed using a scanning electron microscope (LEO1430VP) with an accelerating potential of 15 kV.

### 4.3. Fluorescence Microscopy (FM)

The slides were analyzed under a Nikon Eclipse Ni-U fluorescence microscope without the use of fluorochromes, but with the use of appropriate cut-off filters. The cell wall components were visualized using the autofluorescence method in the fresh material. Lignin and cellulose autofluorescence can be observed using the following filters: an excitation wavelength 330–380 nm and an emission wavelength over 450–480 nm (UV). Lignin shows blue fluorescence while cellulose emits green fluorescence [95]. Photographic documentation was made with a digital camera and NIS-Elements BP software with the use of the EDF module.

### 4.4. Light Microscopy

Seeds were imaged using a Nikon SMZ 74ST stereoscopic microscope, and photographic documentation was made with the use of the Delta Pix program.

### 4.5. Morphometric Measurements

In the group of 25 infructescences located on the main stems and 25 infructescences from the branch stems, the following achene characteristics were measured: total number of achenes (TNA), number of achenes containing a developed embryo (ACE), number of empty achenes (EA), percent share of achenes containing a developed embryo (SACE), receptacle area (RA), and achene density (number of achenes per cm^−2^ of the receptacle area) (AD).

In the other group (25 infructescences located on the main stems (MS) and 25 infructescences located on the branch stems (BS)), each simple infructescence was centrally divided into four sections. The following four categories were distinguished: D1–D3—3 categories of achenes formed from disc florets, and R—one category of achenes formed from peripherally located ray florets. Finally, eight categories were distinguished: MSD1, MSD2, MSD3, MSR, BSD1, BSD2, BSD3, and BSR. From each category, 100 achenes were chosen randomly, and next the following achene characteristics were measured: length of achenes with pappus (LAP), length of achenes without pappus (LA), pappus length (PL), achene width (WA), and achene weight (AW).

### 4.6. Germination Experiment

After conditioning, the achenes were sown into Petri dishes (10 Petri dishes x 8 categories) with moist vermiculite a surface and incubated with simulation of a day/night cycle (16 h light/8 h dark) under light-emitting diodes at photosynthetic photon flux density of 150 μmol m^−2^ s^−1^, temperature regime (25/18 °C), and relative humidity 40–45% for 30 days in a growth chamber. During the experiment, the seeds were watered to maintain constant surface moisture. The experiment lasted 30 days. The germination rate was registered throughout the experiment. When the seedlings formed the first leaf, they were transferred into pots filled with garden soil and placed in a chamber. At the same time, the pericarp was taken, dried, and weighed, and finally the pericarp weight (PW) was measured. The embryo weight (EW) was calculated as the difference between achene weight and pericarp weight and the embryo weight/achene weight ratio (EW/AW) was calculated.

Cultivation was conducted in a growth chamber in the conditions described above for 30 days. In order to conduct the biometrical analysis, 30 randomly selected *A. montana* seedlings from each category (eight achene categories) with two mature leaves were collected. The measurements included the following parameters: seedling weight, seedling stem length, seedling cotyledon length, and seedling root length. The shape index (I) was calculated based on the measurement results of the length and width of the achenes without pappus. The measurements were taken using analytic scales RADWAG, model XA 52.4Y with an accuracy d = 0.01 mg and an electronic caliper ARTPOL with an accuracy of 1 mm.

### 4.7. Statistical Analysis

After testing the data for normality (Shapiro–Wilk test) and homoscedasticity (Levene’s test), analysis of variance of different sets of data was performed, followed by subsequent Tukey’s test or the nonparametric Kruskal–Wallis test, and the subsequent U Mann–Whitney test. In turn, a *t*-test was used to determine if there is a significant difference between the means of two groups. The results were expressed as means and SD, and the differences were considered significant at *p* < 0.05. The statistical analyses were conducted using Statistica 12.0 software (Stat. Soft, Inc., Krakow, Poland). Principal component analysis (PCA) was applied to explain the relationships between the presented parameters and to show the variability of factors. Prior to the PCA, the data were centered, and log transformed. The analyses were conducted using the statistical package (MVSP) program version 3.1 [96].

## 5. Conclusions

The morphological traits of achenes and the reproductive characteristics of *A. montana* were determined by the location of the achene in the infructescence and the location of the inflorescence on the plant. The patterns of the variation in most variables show a decline from the lateral to central positions in the infructescences. The peripherally located achenes were longer with longer pappus, thinner, and were characterized by lower achene weight, lower embryo weight, lower embryo weight/achene weight ratio, and lower germination capacity in comparison to the centrally located achenes. Research on declining arnica populations often requires the use of achenes for propagation, access to which is significantly limited by nature protection institutions for obvious reasons. Therefore, in such situations, achenes located peripherally in the infructescence of the main stem characterized by the greatest mass and germination capacity should be used in propagation, which will contribute to reproductive success. The proposal to use appropriate types of achenes is also relevant for sowing and seedling production. The results presented in this article fill the gap in the knowledge of the morphology of achenes and the biology of the species and provide information that can help in breeding programs, active protection, and field cultivation.

## Figures and Tables

**Table 1:** Results of PCA based on the characteristics of infructescences from the main stem and infructescences from the branch stem. TNA—total number of achenes; ACE—number of achenes containing a developed embryo; EA—number of empty achenes; SACE—percent share of achenes containing a developed embryo; RA—receptacle area; AD—achene density.

	Axis 1	Axis 2
Eigenvalues	2.35	2.26
Percentage	39.21	37.69
Cum. Percentage	39.21	76.90
TNA	0.331	0.57
ACE	0.456	0.452
EA	−0.377	0.43
SACE	0.424	−0.349
RA	0.524	0.062
AD	−0.293	0.401

**Table 2:** Effect of the main factors and their interactions on achene morphometric traits. Results of the two-way analysis of variance (ANOVA). API—the achene position within the infructescence; PIP—the position of the infructescence on the plant; LAP—length of the achene with pappus; LA—length of the achene without pappus; PL—pappus length; WA—width of the achene; TA—thickness of the achene; AW—the achene weight; I—shape index.

	LAP	LA	PL	WA	TA	AW	I
API	F = 268.72	F = 184.64	F = 135.41	F = 7.09	F = 25.54	F = 36.51	F = 40.086
	*p* < 0.001	*p* < 0.001	*p* < 0.001	*p* < 0.001	*p* < 0.001	*p* < 0.001	*p* < 0.001
PIP	F = 202.48	F = 168.77	F = 115.99	F = 19.74	F = 24.19	F = 782.09	F = 0.463
	*p* < 0.001	*p* < 0.001	*p* < 0.001	*p* < 0.001	*p* < 0.001	*p* < 0.001	*p* = 0.496
API × PIP	F = 2.47	F = 0.81	F = 3.07	F = 0.07	F = 0.23	F = 1.76	F = 0.521
	*p* = 0.060	*p* = 0.485	*p* < 0.05	*p* = 0.786	*p* = 0.878	*p* = 0.153	*p* = 0.668

**Table 3:** Effect of the main factors and their interactions on achene characteristics. Results of the two-way analysis of variance (ANOVA). API—the achene position within the infructescence; PIP—the position of the infructescence on the plant; PW—pericarp weight; EW—embryo weight; EW/AW—embryo weight/achene weight ratio; G—germination capacity.

	PW	EW	EW/AW	G
API	F = 1.62	F = 5.74	F = 2.74	F = 6.97
	*p* = 0.185	*p* < 0.001	*p* < 0.05	*p* < 0.001
PIP	F = 101.94	F = 25.78	F = 0.22	F = 1.26
	*p* < 0.001	*p* < 0.001	*p* = 0.639	*p* = 0.265
PIP × API	F = 3.88	F = 2.19	F = 1.39	F = 2.92
	*p* < 0.01	*p* = 0.088	*p* = 0.245	*p* < 0.05

## Data Availability

All the data are available from the corresponding author.
